# Main Human Urinary Metabolites after Genipap (*Genipa americana* L.) Juice Intake

**DOI:** 10.3390/nu10091155

**Published:** 2018-08-24

**Authors:** Livia Dickson, Mathieu Tenon, Ljubica Svilar, Pascale Fança-Berthon, Raphael Lugan, Jean-Charles Martin, Fabrice Vaillant, Hervé Rogez

**Affiliations:** 1 Federal University of Pará & Centre for Valorization of Amazonian Bioactive Compounds (CVACBA), Parque de Ciência e Tecnologia Guamá, Avenida Perimetral da Ciência, km 01, Guamá 66075-750, Brazil; livia_dickson@yahoo.com.br; 2 Naturex SA, 250 rue Pierre Bayle, BP81218, 84911 Avignon CEDEX 9, France; m.tenon@naturex.com (M.T.); p.fancaberthon@naturex.com (P.F.-B.); 3 Centre International de Recherche Agronomique pour le Développement (CIRAD), Avenue Agropolis, TA50/PS4, 34398 Montpellier CEDEX 5, France; fabrice.vaillant@cirad.fr; 4 Aix Marseille Univ, INSERM, INRA, C2VN, CRIBIOM, 5-9, Boulevard Maurice Bourdet, CS 80501, 13205 Marseille CEDEX 01, France; ljubica.svilar@univ-amu.fr (L.S.); jean-charles.martin@univ-amu.fr (J.-C.M.); 5 UMR Qualisud, Université d’Avignon, 301 rue Baruch de Spinoza, BP21239, 84916 Avignon CEDEX 9, France; raphael.lugan@univ-avignon.fr

**Keywords:** biomarker prediction, exposure, high-resolution mass spectrometry, iridoid, phenolic derivatives

## Abstract

Genipap (*Genipa americana* L.) is a native fruit from Amazonia that contains bioactive compounds with a wide range of bioactivities. However, the response to genipap juice ingestion in the human exposome has never been studied. To identify biomarkers of genipap exposure, the untargeted metabolomics approach in human urine was applied. Urine samples from 16 healthy male volunteers, before and after drinking genipap juice, were analyzed by liquid chromatography–high-resolution mass spectrometry. XCMS package was used for data processing in the R environment and *t*-tests were applied on log-transformed and Pareto-scaled data to select the significant metabolites. The principal component analysis (PCA) score plots showed a clear distinction between experimental groups. Thirty-three metabolites were putatively annotated and the most discriminant were mainly related to the metabolic pathways of iridoids and phenolic derivatives. For the first time, the bioavailability of genipap iridoids after human consumption is reported. Dihydroxyhydrocinnamic acid, (1R,6R)-6-hydroxy-2-succinylcyclohexa-2,4-diene-1-carboxylate, hydroxyhydrocinnamic acid, genipic acid, 12-demethylated-8-hydroxygenipinic acid, 3(7)-dehydrogenipinic acid, genipic acid glucuronide, nonate, and 3,4-dihydroxyphenylacetate may be considered biomarkers of genipap consumption. Human exposure to genipap reveals the production of derivative forms of bioactive compounds such as genipic and genipinic acid. These findings suggest that genipap consumption triggers effects on metabolic signatures.

## 1. Introduction

The metabolomics approach is one of the main tools used to characterize the exposome. The human metabolome displays a central role in the study of the human exposome [1]. Therefore, this approach is used in the discovery of biomarkers. [2].

Biomarker discovery after specific external exposure in clinical trials allows the measurement of differences between biological states, including in food exposure studies that seek to determine the impact on human health. The use of these biomarkers provides evidence that includes the dose–response relationship, biotransformation, and correlation with the source of exposure [3,4]. Urine is a widely used biofluid for metabolomics investigations due to the noninvasive collection, the complex metabolic nature of the fluid, and the ability to collect multiple samples over a period of time [5,6]. The permanence of biomarkers of food intake in human urine is around 5–10 h, reaching up to 48 h after ingestion [7].

*Genipa americana* L., genipap, is a native fruit from Amazonia that belongs to the *Rubiaceae* family plant and appears to be a promising source of new bioactive substances for developing new products [8]. Genipap is widely distributed throughout the humid tropics and parts of the subtropical areas of the Americas [9,10,11]. The ripe fruit can be used in different forms: fresh, jams, ice cream, and beverages such as liqueurs, juices, cool drinks, syrups, genipapada, wine, and brandy [12,13,14]. In addition, genipap has been used to treat anemia, uterine cancer, and measles, and it has been used as a diuretic, digestive, laxative, and antiseptic for healing [12,13,15,16,17]. The main compounds reported in genipap belong to the iridoid class. Iridoids are a class of compounds that are of special interest in research and are cyclopenta[c]pyran monoterpenoids with a wide range of bioactivities [18]. Some iridoids from the genipap fruit have already been reported in the literature: genipin, genipic acid, genipinic acid, geniposidic acid, geniposide, genameside A, genameside B, genameside C, genameside D, genipin-gentiobioside, gardenoside, gardendiol, shanzhiside, deacetylasperulosidic methyl ester, genipacetal, genipaol, genipamide, caffeoylgeniposidic acid, *p*-coumaroylgeniposidic acid, feruloyl gardoside, scandoside methyl ester, gardoside, *p*-coumaroylgenipin gentiobioside, and feruloylgenipin gentiobioside [12,14,19,20]. In vitro and animal experiments have shown that these compounds exhibit biological effects such as anticancer [21,22], placental cell regulation [12], neuroprotective [23] immunomodulatory, antioxidant [24,25], anti-inflammatory [26,27], choleretic [28], hepatoprotective, and hypoglycemic [29]. This study explores human exposure to the genipap juice after a single shot of 500 mL. Thus, to identify biomarkers of exposure, we applied the untargeted metabolomics approach followed by statistical filtering to focus on discriminating metabolites.

## 2. Materials and Methods

### 2.1. Ethical Approval and Subject Recruitment

An open-label crossover clinical trial was conducted in strict accordance with the international ethical guidelines for research involving humans established in the Declaration of Helsinki and in compliance with resolution 196/96—of the National Health Council of the Ministry of Health from Brazil CNS/MS. The study was approved by the ethical committee of the Institute of Health Sciences of the Federal University of Para in February of 2016 (Belém, Brazil; ethical ID: 1.436.134). The protocol was explained to each volunteer, and a written version was given and a consent term signed.

Sixteen male volunteers, aged between 18 and 38 years old with a body mass index (BMI, measured in kg/m^2^) between 18.2 and 31.6, were recruited. Individual recruitment was performed using a questionnaire with a medical history, anthropometry, and behavioral questions (addictions, physical activity, and eating habits) before enrollment. Standard blood tests included total, HDL and LDL cholesterols, triglycerides, alkaline phosphatase, aspartate and alanine aminotransferases, fasting glucose, and C-reactive protein.

The inclusion criteria were as follows: healthy free-living men, aged 18–45 years old, presented a stable weight, free of disease, without addictions (smoker, alcoholic, chemical dependence), free of medications and vitamin supplements for the last six weeks before the study, no antibiotics use for three months prior to study, agreed to follow the dietary recommendation, and gave free and full consent to participate.

The following exclusion criteria were used: history of chronic diseases and serious infections, homeostatic disorders, high-performance athletes, ingestion of more than two cups of coffee per day, history of substance abuse (alcohol, tobacco, and narcotics), allergy or intolerance to genipap pulp or juice prior to or during the study, anorexia, bulimia, binge eating disorder and night eating syndrome, and being under a restrictive or special diet (low-calorie, vegetarian, or vegan).

### 2.2. Juice Preparation

All the fruit was purchased from local producers, three in Para and one in Amazonas state. Fruit that was overripe, with a decayed appearance, mechanical and biological deterioration, or loss of original texture, was excluded. Then, the fruit was sanitized with a sodium hypochlorite solution at 200 ppm for 3 min and washed in tap water. The fruits were manually peeled, and the peel and seeds were discarded. The pulp was vacuum-packed and stored at −18 °C until the juice preparation.

One sample of each fruit origin was separated to perform physical–chemical analysis and the results were expressed as the mean value ± standard deviation (Table S1). The equipment and utensils used in the juice preparation followed hygienic conditions for human consumption, and the microbiological analysis of the juice was in accordance with that specified by Brazilian legislation. A few days before starting the study with volunteers, the juice was prepared by thoroughly mixing genipap pulp, water, and white crystallized cane sugar (50:45:5;*w*:*w*:*w*) and stored at −18 °C.

### 2.3. Study Design

A standardized protocol for dietary exposure was applied to minimize the variability between individuals. The volunteers were instructed to not consume alcoholic drinks or fruits and vegetables two days before and during the study, to follow the recommended diet (Table 1), to avoid ingesting a large volume of liquid (more than approximately one liter of water per day), to maintain their usual physical activity, and to not use any medication, vitamins, or other dietary supplements during the test.

**Figure 1:**
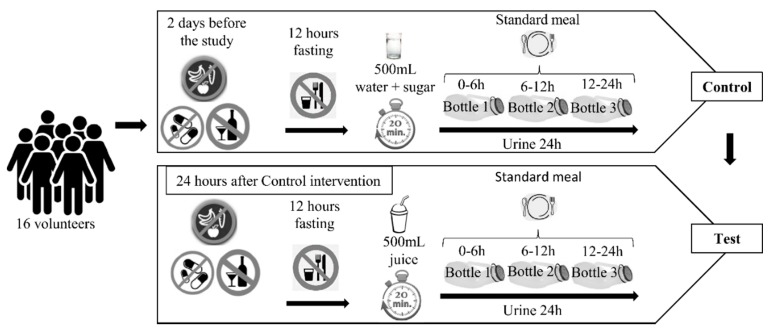
Study design. Sixteen healthy male volunteers were recruited and instructed to follow the recommended diet without alcoholic drinks or fruits and vegetables two days before and during the study. After 12 h fasting, they drank 500 mL of control drink (water + sugar) within a maximum of 20 min and collected the 24 h urine in three different bottles. One day after finishing the control test they started the test with consumption of 500 mL of genipap juice in the same way as the control group.

Two experimental groups were defined including the Control group (before drinking the juice) and the Test group (acute juice ingestion). In the acute juice ingestion study, participants consumed a single 500-mL dose of genipap juice after at least 12 h of fasting. Before drinking, they were asked to empty their bladder and drink the juice within a maximum time of 20 min. All meals during the study were standardized and ingested at specific time points. The 24-h urine samples were collected in three different bottles that correspond to 0–6 h, 6–12 h, and 12–24 h after drinking. The participants were instructed to empty the bladder at the end-point time of each bottle, to start the next one, and to store the samples in the fridge or in Styrofoam boxes with ice. The total volume of each bottle was measured and aliquots were stored at −80 °C. In the control intervention, the volunteers drank 500 mL of water with sugar (the same preparation of genipap juice without the fruit pulp) and the urine samples were collected in the same way (Figure 1).

### 2.4. Sample Preparation

Urine samples were thawed on ice, vortexed for 1 min, and centrifuged at 11,000× *g* for 15 min. at 4 °C. An aliquot of 150 µL of supernatant was diluted with 1.200 µL Milli-Q water and vortexed for 30 s. Samples were centrifuged again at 11,000× *g* for 15 min at 4 °C and the supernatant was kept at −80 °C until the day of analysis. Prior to the analysis, the samples were thawed on ice and vortexed. A pooled sample (pool) used as a quality-control sample (QC) containing equal amounts of all urine samples analyzed was prepared and added to separate vials, for subsequent control of batch drift in the data preprocessing and analysis. A standard mix consisting of l-phenylalanine, l-tryptophan, and creatinine (Sigma-Aldrich, Saint-Quentin-Fallavier, France), at 5 μg/mL was used to provide a fixed reference point of instrumental variation.

### 2.5. UHPLC-HESI-Orbitrap-MS Analysis

Urine samples were analyzed by ultra-high-performance liquid chromatography (Thermo Fisher Scientific, Courtaboeuf, France) using an Hypersil Gold C18 column (100 × 2.1 mm × 1.9 µm) with the temperature set at 40 °C. A gradient elution with acetonitrile containing 0.1% formic acid (A) and water containing 0.1% formic acid (B) was used. The gradient program was as follows: 1 min (100% B), 4.7 min (80% B), 9.5 min (25% B), 11 min (0% B), 12 min (0% B), 13 min (100% B), and 16 min (100% B). The total runtime for each injection was 16 min at a flow rate of 0.4 mL/min. The auto-sampler was conditioned at 4 °C and the injection volume was 5 μL for analysis. For the MS separation, the Q Exactive Focus mass spectrometer (Thermo Fisher Scientific, Courtaboeuf, France) equipped with a heat electrospray ionization (HESI) was used. The spray voltage was set at 3500 V, and the HESI probe and transfer capillary temperature were kept at 310 °C and 320 °C, respectively. The sheath and auxiliary gas were maintained at 30 and 8 (arbitrary units), with the S-lens RF at 55 V. The analysis was performed in full MS scan with switching ionization polarity mode. The resolution was set to 35,000 full widths at half maximum (FWMH) for the *m*/*z* 200. The automatic gain control (AGC) was set at 1e6 with a maximum injection time (IT) of 250 ms. The full MS spectra were acquired in the *m*/*z* range from 80 to 1000 *m*/*z*. All data were collected in profile mode. Samples were analyzed in three batches. Each batch started with five blank sample (deionized water) injections and then 10 QC samples for the chromatographic system equilibration. Then, every fifth randomly chosen experimental sample was followed by one QC sample injection from a separated vial. The ionization source was cleaned every 80 injections in order to diminish analytical drifts.

### 2.6. Preprocessing and Pretreatment of Data

The raw data were converted to positive and negative separated mzXML files in the R environment [30] using the ProteoWizard [31] for data preprocessing. The XCMS package was used for data processing to extract metabolic features from LC-MS data [32] in the R environment. The xcmsSet was used with the centWave method to perform feature extraction by peak detection. Peak detection was carried out using a minimum and maximum peak width of 2 and 15 s, respectively, a snthresh = 3, difference of *m*/*z* at 0.00005 *m*/*z*, the maximal tolerated *m*/*z* deviation in consecutive scans at 5 ppm, and noise threshold was set to 1000 and integration of the *m*/*z* centroid peaks through descent on the Mexican hat-filtered data. For the peak alignment, the Obiwarp method was used, and for grouping the peaks from different samples, a density method was applied. The missing data points were filled by re-reading the mzXML data files and integrating them in the regions of missing peaks using fillPeaks. To perform feature filtration, we used blank samples and coefficient of variation (CV = 0.2, positive mode ionization; CV = 0.3, negative mode ionization).

### 2.7. Data Analysis and Multi-Metabolite Biomarker Model

The univariate and multivariate analyses were performed using Metaboanalyst 3.5 [33] to explore differences between sample groups in both ionization polarity data. Student’s *t*-test, a parametric univariate hypothesis, was used for analysis, and differences with *p*-value adjusted for false discovery rate (FDR) < 0.05 were considered significant. The principal components analysis (PCA) and partial least squares discriminant analysis (PLS-DA) were also performed on auto-scaled data to highlight experimental differences between groups. The annotated features were submitted to PLS-DA analysis using SIMCA P + 12 software (Sartorius, Aubagne, France), and the compound clusters with the most common metabolomic characteristic of the consumers/non-consumers (“c” vector of SIMCA algorithm) were chosen to calculate a predictive equation using the PLS algorithm, as described previously [34]. The variables were putatively annotated using the chemical formula calculated from the exact mass and the MS/MS spectra. The accuracy and sensitivity of this multiple biomarkers of genipap exposure were further assessed using the area under the curve (AUC) of the receiver operator characteristic (ROC) curve in MetaboAnalyst [33].

### 2.8. Annotation, Identification, and Interpretation

The 50 most discriminant features (*p*-value < 0.05 and variable importance on the projection—VIP > 1) in each ionization polarity were tentatively identified as follows. Features with the same retention time, graph behavior and intensity across the samples were grouped in a cluster corresponding to one potential metabolite. Features were selected as precursor ions and fragmented with different normalized collision energies (NCE) namely, 20, 30, and 40 V, and the parent *m*/*z* and the *m*/*z* of the most intense fragments were tentatively annotated based on the compound identification levels suggested by the Metabolomics Standards Initiative [35]. Features were matched with the in-house database using Galaxy platform [36], and the fragmentation pattern was compared to online metabolite databases, including the Human Metabolome Database [37], METLIN [38], MetFrag [39] and MetFusion [40]. Available tools for the functional and biological interpretation [41] as well as reported information in the literature, KEGG encyclopedia [42], and MetaCyc [43] were used.

## 3. Results

### 3.1. Data Treatment and Analysis

**Figure 2:**
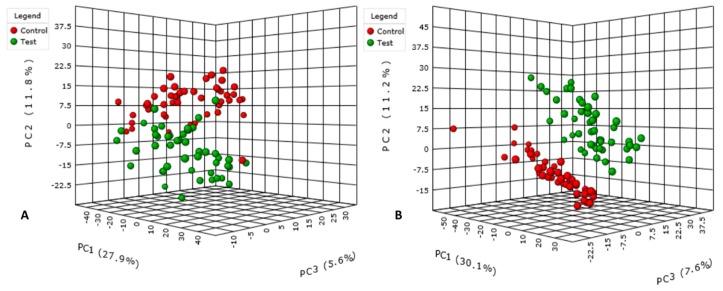
PCA 3D score plot analysis of human urine before (Control) and after (Test) consumption of genipap juice in positive (**A**) and negative (**B**) mode. The analysis was performed using MetaboAnalyst 3.5.

Data preprocessing gave 4038 and 2502 features in positive and negative ionization modes, respectively. After data pretreatment, 1051 features remained in positive and 975 in negative mode. The number of discriminant features remaining after the statistical analysis was 130 and 178 for each polarity, respectively. PCA score plots of both positive (Figure 2A) and negative (Figure 2B) ionization modes highlighted separation between the two groups, control and test.

### 3.2. Annotation and Tentative Identification

**Figure 3:**
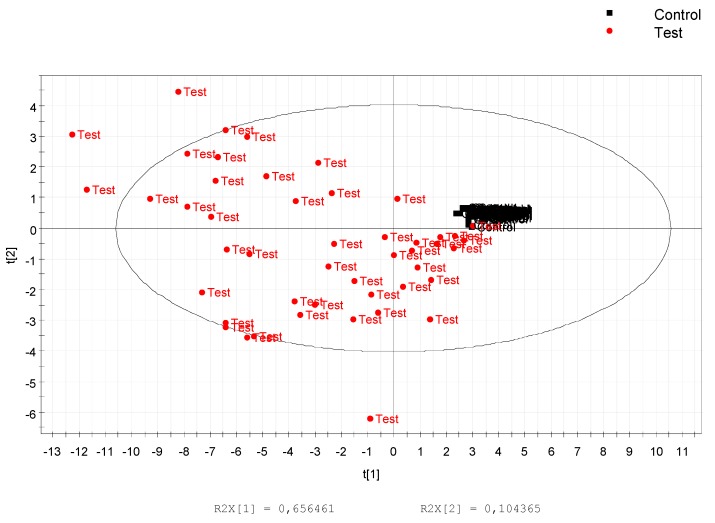
PLS-DA score of 34 noted features performed using SIMCA P12 software. The distribution shows a significant difference and individual variation after drinking the juice.

**Figure 4:**
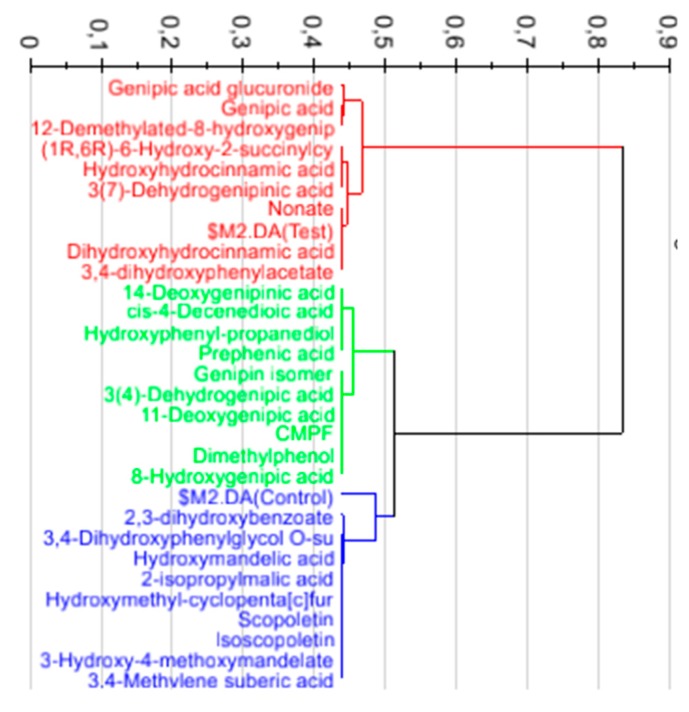
Hierarchical clustering analysis of the PLS-DA loadings showing the metabolites most closely clustering with the consumers’ ($M2.DA (Test in red)), the group of metabolites less well associated with the control (in green), and non-consumers’ metabolome ($M2.DA (Control in blue)).

The 50 most significant features in each ionization mode were putatively annotated in four different levels according to the Metabolomic Standards Initiative [27] (Tables S2 and S3). A total of 33 features were putatively annotated at level III or below in both ionization polarities. PLS-DA analysis using only annotated features allowed enhancement of the significant differences between groups before and after drinking the juice (Figure 3). In addition, the PLS-DA allowed for selecting nine of the most discriminant features based on the best correlation to the metabolomic score specific to genipap consumers (Figure 4).

### 3.3. Calculation and Validation of a Multiplex Biomarker of Genipap Exposure

The nine most discriminating annotated metabolites were combined into a predictive equation using the PLS algorithm of SIMCA, as described [38]. To assign with confidence, the genipap consumers before and after consumption, the predictive equation was performed (Equation (1)), where the metabolite urine content was expressed in % of the sum of the nine compounds.

pred (Test) = 0.0033562 × [% Dihydroxyhydrocinnamic acid] − 0.00462195 × [% (1R,6R)-6-Hydroxy-2-succinylcyclohexa-2,4-diene-1-carboxylate] − 0.0102557 × [% Hydroxyhydrocinnamic acid] − 0.144505 × [% Genipic acid] + 0.235765 × [% 12-Demethylated-8-hydroxygenipinic acid] + 0.00399005 × [% 3(7)-Dehydrogenipinic acid] + 0.00433891 × [% Genipic acid glucuronide] − 0.0110051 × [% Nonate] − 0.0174746 × [% 3,4-dihydroxyphenylacetate] + 0.634178(1)

**Figure 5:**
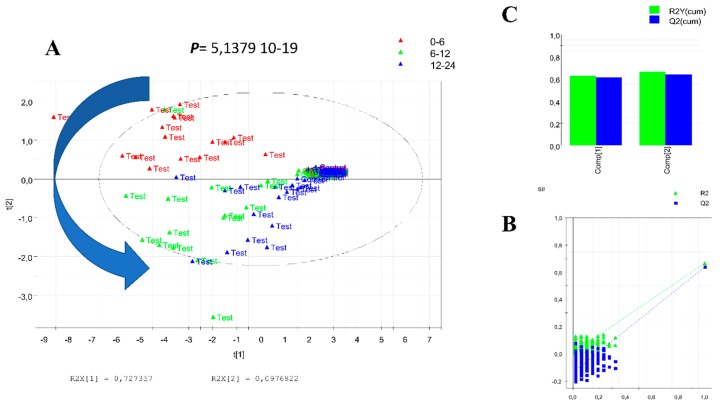
PLS-DA analysis of nine of the most discriminant features in two conditions. (**A**) PLS-DA model among control and test shows very significant (1.64587 × 10−22) difference between classes. (**B**) Permutation test, method validation R2 = (0.0, 0.00525), and Q2 = (0.0, −0.13). (**C**) Two groups’ PLS-DA score and the predictive model cutoff.

A PLS-DA model was first constructed using this combination and was strongly validated, with the cross-validation ANOVA *p*-value (5.14 × 10^−19^), the R2Y and Q2Y values prior (0.672 and 0.668, respectively) and after permutation (−0.022 and −0.098, respectively), demonstrating no overfitting (Figure 5).

**Figure 6:**
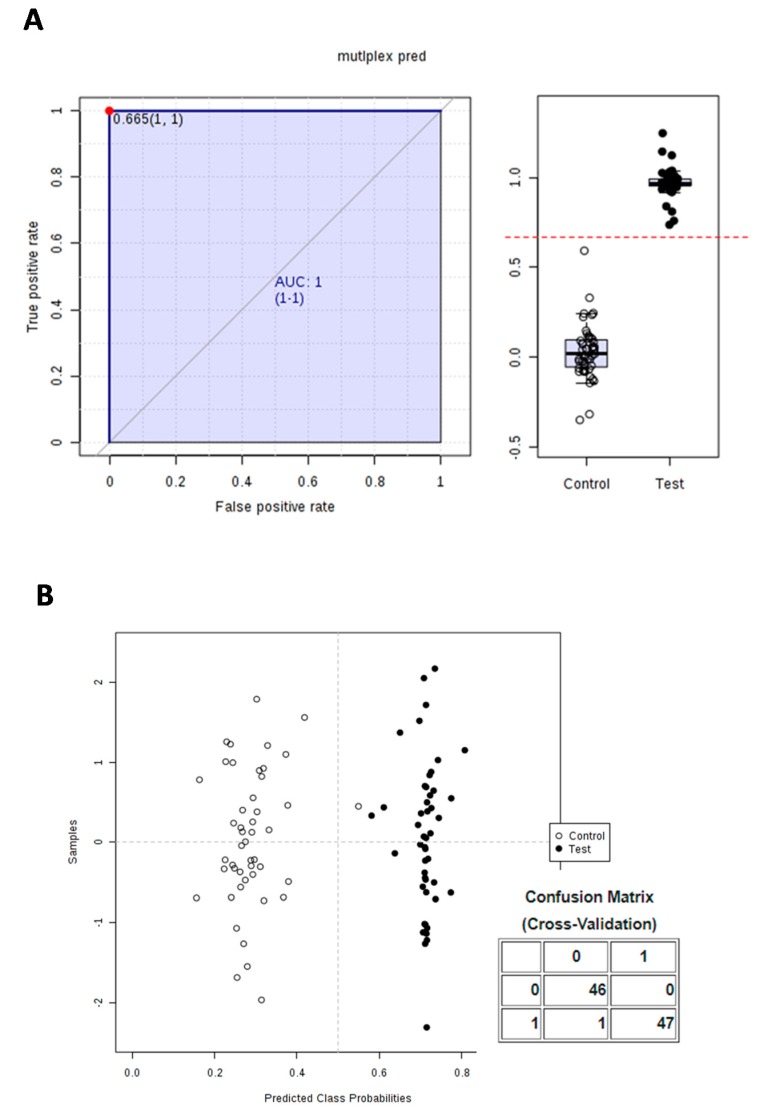
ROC curve analysis. (**A**) Diagram of two components (control and test) with excellent value (AUC = 1; CI = 1^−1^), cross-validations and result expressed as averaged to generate the plot. (**B**) Predicted class probabilities calculated from the ROC curve, along with the confusion matrix obtained after cross-validation and showing only one misclassified individual.

In addition, the prediction score was also challenged using the ROC procedure. ROC analysis was applied to evaluate the performance of the model and to determine the optimal threshold (cut-off value) beyond which features could allow the classification of individuals as control or test. ROC analysis is a standard way to describe the accuracy of a diagnostic test where the results are recorded as dichotomous outcomes (positive/negative results) and is broadly applied for medical diagnostic test evaluation [44]. Using all samples collected over 24 h, an ROC diagram of two components (control and test) with excellent value (AUC = 1; CI = 1^−1^) [44] is observed in Figure 6A. Figure 6A shows the predicted class probabilities for each sample using the best classifier based on AUC. An error rate of 1/93 was observed (Figure 6B), with a cut-off value of 0.665 for distinguishing consumers from non-consumers (Figure 6A). This result indicates that an individual scoring less than 0.665 in the equation can be considered as being before consumption, and the reverse when the score is over that cut-off.

To challenge the prediction power of the multiplexed biomarker defined over a 24-h period, PLS-DA models were calculated at each time range of urine collection (e.g., 0–6 h, 6–12 h, and 12–24 h) and were all found to be highly significantly discriminant (*p* = 4.23 × 10^−9^, 2.5 × 10^−7^ and 0.0019, respectively, not shown). The robustness of the multiplexed biomarker was also assessed using ROC analysis, using all the samples collected over 24 h, or stratified over each time-range of collection (0–6 h, 6–12 h, and 12–24 h). The multiplexed biomarker was as good as the best individual predictive metabolites (AUC of 1) at each time point (Table 2).

Finally, when compared to individual metabolites occurring specifically from genipap fruit over all the time frames, the multiplex biomarker appeared less susceptible to inter-individual variations to predict the genipap consumer status (Figure S3).

## 4. Discussion

In this study, we used the untargeted LC-MS metabolomics to identify biomarkers excreted in 24 h urine before and after consumption of a single dose (500 mL) of genipap juice. We annotated 34 metabolites from the 100 most discriminant features in both ionization modes.

Fifteen metabolites characterized at level III or below were from the iridoid family and some of them were also identified in genipap juice. For this class of compounds, we were not able to perform identification at level I because reference standards were not commercially available.

Genipin is an aglycone derived from geniposide that can be hydrolyzed to genipin by β-glucosidases of intestinal bacteria [45]. Genipin is the iridoid mostly present in genipap reported in the literature. Studies reported bioactivities of genipin as anticancer [21,22,46], anti-inflammatory [27,47], immunomodulatory [18,24], choleretic [28,48], and antioxidant [18,25]. To our knowledge, this is the first report about genipin’s bioavailability, as we could identify it in urine samples. The identification of genipin in urine was performed by comparison of the fragmentation spectra to those of a commercial standard of genipin (Figure S4). The spectra fragmentation of commercial standard was similar to the fragmentation in urine, but a different retention time was observed, and thus we assumed that the compound present in urine after genipap oral ingestion is an isomer.

**Figure 7:**
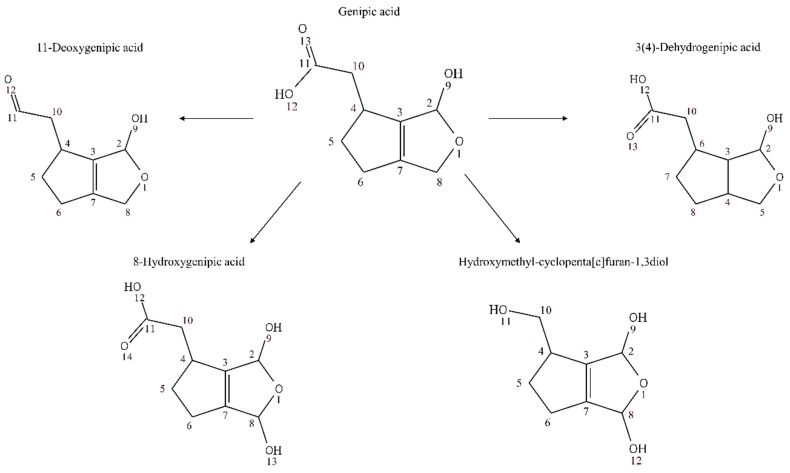
Proposed derivative forms of genipic acid in human urine samples after genipap juice intake.

**Figure 8:**
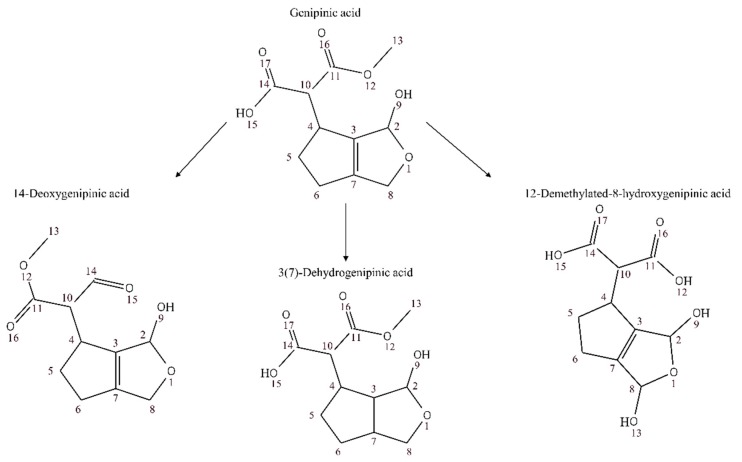
Proposed derivative forms of genipinic acid in human urine samples after genipap juice intake.

We detected derivative forms of genipic acid (Figure 7) and genipinic acid (Figure 8) in urine samples: 14-deoxygenipinic acid, 11-deoxygenipic acid, 3(4)-dehydrogenipic acid, 11-Deoxygenipic acid isomer, 12-Demethylated-8-hydroxygenipinic acid, 3(7)-dehydrogenipinic acid, 3(4)-dehydrogenipic acid isomer, 3(7)-dehydrogenipinic acid isomer, 8-Hydroxygenipic acid, hydroxymethyl-cyclopenta[c]furan-1,3-diol, genipic acid glucuronide and genipic acid isomer. The genipic acid was detected in both ionization modes at level III ([M + H]^+^, *m*/*z* 185.0808, retention time: 5.38; [M − H]^−^, *m*/*z* 183.0664, retention time: 5.39). Genipic acid and genipinic acid, were first described as antibiotic cyclopentanoid monoterpenes isolated from *Genipa americana* L., which were able to inhibit the in vitro growth of a wide variety of Gram-negative and Gram-positive bacteria, a fungus (*Trichophyton menrugruphytes*), an algae (*Chlorella uulguris*), and a protozoan (*Tetruhymena gelleii*) [49].

Our findings show that consuming genipap juice allows large exposure of the body to iridoids, especially derivatives of genipic and genipinic acid.

No data about the bioavailability of iridoids in humans have been reported until now. Almost all investigations are in vitro or in an animal model. Our results show for the first time the bioavailability of some iridoids from genipap juice after human consumption. The iridoids detected in human urine may result from microflora metabolism and phase II biotransformation reactions. All the previous tentatively identified metabolites from the iridoid family were detected in the urine of all volunteers after oral ingestion of genipap juice; they were not detected at all in urine before ingestion.

Ueda et al. [50] demonstrated the anti-tumor activity of iridoids from fruits and leaves of genipap in cell cultures. Finco et al. [51] tested the antiproliferative effects of genipap extracts and the result was a significant inhibition of human liver cancer cell line (HepG2 cell) proliferation in a dose-dependent manner. In addition, the extract had no effect on BeWo cell (human trophoblastic endocrine cell type) differentiation through human chorionic gonadotrophin release or syncytial formation. However, *G. americana* fruit extract has influenced the cell-signaling pathway and inhibition of trophoblast-like cell proliferation.

We identified 4 phenolic derivatives: dihydroxyhydrocinnamic acid, hydroxyhydrocinnamic acid, scopoletin and 3,4-dihydroxyphenylacetate. Dihydroxyhydrocinnamic acid, hydroxyhydrocinnamic acid and 3,4-dihydroxyphenylacetate are produced by bacterial degradation in the intestine and colon and possess antioxidant and antiproliferative activities [42,43,52,53]. Scopoletin is a coumarin that is widespread in the plant kingdom and has diverse pharmacological properties as an anti-inflammatory, and antitumor and radical-scavenging activities [43].

Additionally, an intermediate of vitamin K biosynthetic pathway was identified, (1R,6R)-6-Hydroxy-2-succinylcyclohexa-2,4-diene-1-carboxylate ([M + H]^+^, *m*/*z* 241.0703, retention time: 4.93). The biosynthetic pathway of vitamin K also leads to the biosynthesis of many plant pigments, mostly found in the Rubiaceae family and involved in plant defense. Vitamin K plays a key role in blood coagulation and has an important role in the prevention and treatment of bone and vascular disease [43,54].

The 3-carboxy-4-methyl-5-propyl-2-furanpropanoic acid (CMPF), a furan dicarboxylic acid derivative, was identified at level II in positive mode ([M + H]^+^, *m*/*z* 241.1068, retention time: 4.62). One can speculate that CMPF is a product of microbiome activity. The furan dicarboxylic acids have some reported bioactivities such as antioxidant, anti-inflammatory, and inhibition of non-enzymatic lipid peroxidation. There is some controversy about the possible association of increased CMPF with type 2 diabetes and renal failure, since the measurement of the research results is not standardized and can lead to misinterpretation [55].

Our data prove the presence of bioactive compounds from genipap juice in human urine. All non-iridoid metabolites that we identified had their intensities increased after the ingestion of the genipap juice. The inter-individual variation after drinking the juice (Figure 3) may be a result of genetic determinants, absorption mechanism, and microflora metabolism. As we have putatively identified metabolites in urine that were already shown in the literature to have high bioactivity in vitro, it can be suggested that oral ingestion of genipap may produce potentially the same health effects, such as antioxidant, anti-inflammatory, and healing.

We selected and annotated nine urine metabolites indicative of genipap intake over 24 h. The prediction was more accurate after the early phase of consumption than later on, but still remained high in the late phases (12–24 h). Some of these metabolites (12-demethylated-8-hydroxygenipinic acid, genipic acid glucuronide, 3(7)dehydrogenipinic acid, and genipic acid) are specific to genipap and were, indeed, individually strongly predictive of genipap intake at any time up to 24 h. However, the cut-off values mainly rely on instrument response and would require an absolute quantification. Such quantification is not trivial in urine, since it requires scaling to a reference value (such as creatine, urine volume, and urine density), which are not absolute. Measurement instability can thus generate biases in classification. Hence, it can be more advantageous to rely on several biomarkers rather than single ones, not only for specificity and sensitivity reasons and to improve accuracy [56] but also to avoid the absolute quantification. In fact, our multiplex classifier ranged from 0 (control individuals) to 1 (after consuming genipap). It is calculated irrespective of the absolute concentration of metabolites in urine. More precisely, this classifier relied on the relative proportion of its constitutive metabolites (% values among the nine selected metabolites) and constitutes a pattern with no quantitative reference. Such a pattern makes our classifier less prone to inter-individual variations than individual metabolites by itself (Figure 3).

## 5. Conclusions

The multiplex biomarker model allows for determining if an individual has consumed genipap juice recently, and these data can be used in epidemiological studies to help understand its use to treat some diseases in the Amazon regions. In addition, this model may be applied to test products containing genipap. Furthermore, it could be an interesting strategy to design new biomarkers with increased specificity and sensitivity. It would also be interesting to assess how this multiplex biomarker can report individuals with various genipap intake levels, and thus infer the fruit exposure dose–response relationship. Further analysis to decipher the mechanisms of absorption and the dose–response relationship of bioactive compounds present in genipap are ideas for future research.

## Figures and Tables

**Table 1:** Recommended diet, excluding: Whole food products (bread, cereals, flour, biscuits, etc.) nuts and seeds, almonds, fruit and fruit-containing products, vegetables, chocolate, alcoholic beverages, tea, and coffee.

Meal	Food
Breakfast	Bread with butter, or bread with cheese, or bread with cheese and ham, or toast with butter, or cream crackers + yogurt *
Snack	Cream crackers + yogurt *
Lunch	Rice or pasta + beans + grilled (or roasted) chicken or meat + mashed potatoes Dessert: gelatin
Snack	Sandwich without salad + yogurt or other beverage *
Dinner	Pasta

* That does not contain fruit or whole grains.

**Table 2:** Performance of selected metabolites at each time period as assessed by ROC analysis.

Time Range	Metabolites	AUC	*p*-Value (*t*-Test)	Cut-Off Value
All times	1R,6R-6-Hydroxy-2-succinylcyclohexa-2,4-diene-1-carboxylate	1	2.0454 × 10^−21^	5
	Hydroxyhydrocinnamic acid	1	5.7497 × 10^−28^	2.64
	multiplex pred	1	6.8817 × 10^−57^	0.665
	3,4-dihydroxyphenylacetate	0.99593	1.35 × 10^−14^	0.63
	3(7-dehydro)genipinic acid	0.98008	7.1576 × 10^−14^	4.3
	Nonate	0.97737	3.9231 × 10^−17^	2.47
	12-demethylated-8-hydroxygenipinic acid	0.95292	5.7703 × 10^−7^	0.00878
	Dihydroxyhydrocinnamic acid	0.93074	3.0562 × 10^−18^	37.8
	Genipic acid	0.85785	1.0072 × 10^−9^	0.322
	Genipic acid glucuronide	0.74423	0.55998	0.218
0 to 6 h	Dihydroxyhydrocinnamic acid	1	1.9091 × 10^−14^	48.9
	1R,6R-6-Hydroxy-2-succinylcyclohexa-2,4-diene-1-carboxylate	1	9.9186 × 10^−10^	3.95
	Hydroxyhydrocinnamic acid	1	7.7957 × 10^−12^	3.56
	Nonate	1	8.2316 × 10^−9^	5.85
	3,4-dihydroxyphenylacetate	1	1.6602 × 10^−7^	0.244
	multiplex pred	1	1.3766 × 10^−22^	0.633
	Genipic acid	0.98333	7.4173 × 10^−8^	0.342
	3(7-dehydro)genipinic acid	0.95	5.951 × 10^−7^	4.14
	12-demethylated-8-hydroxygenipinic acid	0.9375	2.574 × 10^−4^	0.0159
	Genipic acid glucuronide	0.93333	2.0461 × 10^−4^	0.17
6 to 12 h	1R,6R-6-Hydroxy-2-succinylcyclohexa-2,4-diene-1-carboxylate	1	6.1416 × 10^−9^	4.83
	Hydroxyhydrocinnamic acid	1	1.7482 × 10^−9^	2.64
	12-Demethylated-8-hydroxygenipinic acid	1	6.9629 × 10^−5^	0.00947
	Nonate	1	9.5177 × 10^−6^	3.09
	3,4-dihydroxyphenylacetate	1	1.1069 × 10^−4^	0.892
	multiplex pred	1	2.2156 × 10^−21^	0.486
	3(7-dehydro)genipinic acid	0.97917	1.3988 × 10^−5^	3.5
	Dihydroxyhydrocinnamic acid	0.93333	3.7546 × 10^−6^	36.8
	Genipic acid	0.77083	0.0073173	0.322
	Genipic acid glucuronide	0.7625	0.92915	0.374
12 to 24 h	1R,6R-6-Hydroxy-2-succinylcyclohexa-2,4-diene-1-carboxylate	1	6.279 × 10^−6^	6.12
	Hydroxyhydrocinnamic acid	1	2.1664 × 10^−10^	3.85
	3,4-dihydroxyphenylacetate	1	9.8555 × 10^−6^	0.63
	multiplex pred	1	1.4956 × 10^−16^	0.665
	3(7-dehydro)genipinic acid	0.99219	1.2466 × 10^−5^	1.74
	12-demethylated-8-hydroxygenipinic acid	0.92929	0.002416	0.0106
	Nonate	0.92578	1.7992 × 10^−5^	7.13
	Dihydroxyhydrocinnamic acid	0.86528	1.8911 × 10^−4^	50
	Genipic acid	0.83984	8.338 × 10^−4^	0.318
	Genipic acid glucuronide	0.55469	0.22484	0.242

The multiplex biomarker was steady across each time range, more than any of its individual constituent (Figure S1, Venny plot). In addition, in order to assess if the multiplex biomarker predictive scores determined at a selected time range can be used any time over 24 h, we permuted the threshold of each ROC model to determine the impact on genipap consumers and non-consumers’ status determination. The prediction was perfect until 12 h and was only minimally affected in the range 12–24 h, with one misclassified individual (Figure S2).

## Supplementary Materials

The following are available online at http://www.mdpi.com/2072-6643/10/9/1155/s1, Table S1: Physical chemical analysis in genipap pulp, Table S2: 50 (*p*-value < 0.05) most discriminant features in positive mode, Table S3: 50 (*p*-value < 0.05) most discriminant features in negative mode, Figure S1: Common and more specific biomarker of genipap consumption at each time-range. The intersection indicates the steadiest biomarkers across each time range. Multiplex Pred indicates the predictive multiplex biomarker, Figure S2: Genipap consumers’ vs. non-consumers’ prediction using the urine multiplex biomarkers measured at 0–6 h, 6–12 h and 12–24 h. The cut off values indicating genipap consumption status were permuted to assess the impact of the sampling time on the prediction over 24 h. The actual cut-off values of each time range are indicated in red. Misclassified samples are indicated with an arrow, Figure S3: Inter-individual variation of best predicting metabolites (ROC AUC of 1, Table 2) at 0–6 h (A), 6–12 h (B), and 12–24 h (C) urine compared to the multiplex biomarker scores (yellow line) calculated over the same time ranges. All values are scaled to 1 to ease comparisons, Figure S4: MS/MS spectra fragmentation. (A) spectrum of commercial standard of genipin, (B) spectra of a metabolite obtained from human urine sample after drinking genipap juice ([M + H]^+^, *m*/*z* 227.0911, retention time: 4.04).
