# Supplementary material for: Main Human Urinary Metabolites after Genipap (Genipa americana L.) Juice Intake
Source: Nutrients. 2018 Aug 24;10(9):1155. doi: 10.3390/nu10091155 (PMC6165415; doi:10.3390/nu10091155)

Supporting Information

Table S1: Physical chemical analysis in genipap pulp.

Physical chemical parameters	Mean and standard deviation
Ash (%)	3.26 ± 0.17
Titratable acidity (g Eq. citric acid/100 g)	0.85 ± 0.26
pH	3.95 ± 0.14
Moisture (%)	77.32 ± 0.60
Total soluble solids (ºBRIX)	20.32 ± 0.58
Total phenolics (mg Eq. galic acid/g)	91.71 ± 3.10
ORAC[a] (µmol Eq. Trolox/100 g)	6.71 ± 0.82
Total flavanols (mg Eq. catechin/g)	0.03 ± 0.00
Total flavonols (mg Eq. rutin/kg)	0.54 ± 0.12
Ascorbic acid mg/100 g	0.19 ± 0.06
Carbohydrates (%)	9.16 ± 2.64
Fiber (%)	7.73 ± 1.88
Proteins (%)	2.04 ± 1.09
Lipides (%)	0.67 ± 0.15

[a] Oxygen radical absorbance capacity

Table S2. 50 (P-value < 0.05) most discriminant features in positive mode.

Annotated metabolite	MS Main ions *m/z* (*p*-value classification)	Ion	RT (min)	Proposed precursor ion formula	Mass precision (ppm)	MS/MS (*m/z*) main fragments (30-40 v)	Reference/ identification level
Genipin isomer	227.0911 (16)<br>209.0807 (1)<br>165.0909 (4)<br>163.0751 (14)<br>149.0596 (5)	$[M + H]^+$<br>$[M - H_2O]^+$	4.04	$C_{11}H_{14}O_5$	- 0.9	209.0807<br>163.0751<br>149.0596<br>105.0697	Putatively characterized compound classes (III)
Dihydroxyhydrocinnamic acid	183.0650 (2)<br>184.0682 (27)<br>165.0544 (28)	$[M + H]^+$<br>$[[M + H]+^{13}C]^+$<br>$[M - H2O]^+$	4.84	$C_9H_{10}O_4$	- 1.0	165.0544<br>137.0595	HMDB ID: HMDB0000423 (II)
11-Deoxygenipic acid	169.0858 (15)<br>151.0752 (3)<br>107.0853 (10)	$[M + H]^+$<br>$[M - H_2O]^+$<br>$[M - CO_2]^+$	3.89	$C_9H_{12}O_3$	-1.0	151.0752<br>107.0853	Putatively characterized compound classes (III)
(1R,6R)-6-Hydroxy-2-succinylcyclohexa-2,4-diene-1-carboxylate	241.0703 (45)<br>223.0598 (6)	$[M + H]^+$<br>$[M - H_2O]^+$	4.93	$C_{11}H_{12}O_6$	- 1.3	223.0598<br>209.0440<br>195.0643<br>177.0542<br>119.0489	KEGG ID: C05817 (III)
3(4)-Dehydrogenipic acid	187.0962 (7)	$[M + H]^+$	3.87	$C_9H_{14}O_4$	- 1.5	169.0856<br>151.0751<br>107.0852	Putatively characterized compound classes (III)
14-Deoxygenipinic acid	227.0912 (8)<br>210.0839 (32)<br>209.0807 (11)<br>163.0752 (31)<br>149.0596 (18)<br>135.0803 (30)	$[M + H]^+$<br><br>$[M - H_2O]^+$	4.68	$C_{11}H_{14}O_5$	- 0.7	209.0807<br>191.0699<br>177.0543<br>163.0750<br>149.0595<br>135.0802<br>121.0646<br>93.0697	Putatively characterized compound classes (III)
Genipic acid glucuronide	378.1394 (9)	$[M + H]^+$	4.79	$C_{15}H_{20}O_{10}$	0.8	185.0807<br>167.0698<br>139.0751<br>95.0853	(IV)

Table S2. continued

Annotated metabolite	MS Main ions *m/z* (*p*-value classification)	Ion	RT (min)	Proposed precursor ion formula	Mass precision (ppm)	MS/MS (*m/z*) main fragments (30-40 v)	Reference/ identification level
Unknown	228.1229 (12)		4.84			183.0649	(IV)
Dihydroxyhydrocinnamic acid isomer	183.0650 (29)<br>165.0544 (13)<br>137.0596 (25)	$[M + H]^+$<br>$[M - H_2O]^+$<br>$[M - HCOOH]^+$	4.06	$C_9H_{10}O_4$	- 0.8	165.0544<br>137.0596	HMDB ID: HMDB0000423 (II)
CMPF (3-carboxy-4-methyl-5-propyl-2-furanpropanoic acid)	241.1068 (21)<br>223.0962 (17)	$[M + H]^+$	4.62	$C_{12}H_{16}O_5$	- 0.7	223.0974<br>196.1692<br>181.0861	HMDB ID: HMDB0061112 (II)
11-Deoxygenipic acid isomer	169.0494 (19)	$[M + H]^+$	4.15	$C_8H_8O_4$	- 0.3	151.0751<br>107.0852	Putatively characterized compound classes (III)
Unknown	225.0755 (20)		4.58			207.0646<br>189.0543<br>161.0594<br>151.0388<br>119.0490	(IV)
Dimethylphenol	123.0803 (22)	$[M + H]^+$	3.91	$C_8H_{10}O$	-0.9	105.0696<br>95.0853<br>81.0696<br>67.0540	METLIN ID: 88228 (III)
Hydroxyhydrocinnamic acid	167.0700 (23)	$[M + H]^+$	1.79	$C_9H_{10}O_3$	-1.3	149.0132<br>121.0644<br>107.0489	HMDB ID: HMDB00375 (III)
Hydroxyphenyl-propanediol	169.0858 (24)	$[M + H]^+$	4.77	$C_9H_{12}O_3$	-0.7	151.0751<br>123.0802<br>107.0852	METLIN ID: 984984 (II)
Unknown	169.0494 (26)		3.49			151.0751<br>124.0867<br>107.0853<br>100.0754<br>94.0285	(IV)

Table S2. continued

Annotated metabolite	MS Main ions *m/z* (*p*-value classification)	Ion	RT (min)	Proposed precursor ion formula	Mass precision (ppm)	MS/MS (*m/z*) main fragments (30-40 v)	Reference/ identification level
Unknown	167.0702 (33)		2.22			149.0134<br>121.0646	(IV)
Prephenic acid	227.0549 (40)<br>209.0443 (34)	$[M + H]^+$<br>$[M - H_2O]^+$	1.82	$C_{10}H_{10}O_6$	-0.8	209.0442<br>191.0334<br>163.0386<br>137.0596<br>135.0438	HMDB ID:<br>HMDB0012283 (III)
Unknown	197.0806 (35)		5.32			190.9925<br>141.0002	(IV)
Scopoletin	193.0494 (36)	$[M + H]^+$	5.87	$C_{10}H_8O_4$	- 0.6	175.1471<br>147.0437	HMDB ID:<br>HMDB0034344 (II)
Genipic acid	185.0808 (37)<br>186.0840 (44)<br>139.0753 (41)<br>95.0853 (47)	$[M + H]^+$<br>$[[M + H]+^{13}C]^+$<br>$[M - HCOOH]^+$	5.38	$C_9H_{12}O_4$	-0.0	167.0700<br>139.0753<br>95.0853	METLIN ID:<br>91245<br>HMDB ID:<br>HMDB0036072 (III)
Isoscopoletin	193.0494 (38)	$[M + H]^+$	5.28	$C_{10}H_8O_4$	- 0.6	175.0387<br>147.0439	KEGG ID:<br>C18079 (III)
Unknown	137.0597 (39)		4.82				(IV)
Unknown	209.0807 (42)		5.32			177.0544<br>149.0595<br>141.0002<br>131.0490<br>105.0696	(IV)
Unknown	199.0964 (43)		5.58			169.9770<br>135.0802	(IV)
Unknown	155.0701 (46)		2.60			137.0594<br>153.0907<br>135.0805<br>125.0595<br>107.0853<br>89.0707	(IV)
Unknown	171.1013 (48)		4.31			153.0907<br>125.0595	(IV)

Table S2. continued

Annotated metabolite	MS Main ions *m/z* (*p*-value classification)	Ion	RT (min)	Proposed precursor ion formula	Mass precision (ppm)	MS/MS (*m/z*) main fragments (30-40 v)	Reference/ identification level
Unknown	373.1489 (49)		5.72			197.1164<br>179.1062	(IV)
Unknown	292.1571 (50)		5.73			233.0837<br>103.0573	(IV)

Levels of metabolite identifications: I, Identified compounds; II, Putatively annotated compounds; III, Putatively characterized compound classes; IV, Unknown compounds.

Table S3. 50 (P-value < 0.05) most discriminant features in negative mode.

Annotated metabolite	MS Main ions *m/z* (*p*-value classification)	Ion	RT (min)	Proposed precursor ion formula	Mass precision (ppm)	MS/MS (*m/z*) main fragments (30-40 v)	Reference/ identification level
12-Demethylated-8-hydroxygenipinic acid	243.0510 (7)<br>244.0545 (13)<br>199.0613 (9)<br>155.0714 (1)	$[M - H]^-$<br>$[[M-H]+^{13}C]^-$<br>$[M - CO_2]^-$	1.82	$C_{10}H_{12}O_7$	0.0	199.0613<br>155.0714<br>137.0607	Putatively characterized compound classes (III)
Unknown	229.0718 (2)		3.13			211.0611<br>185.0818<br>150.0559	(IV)
3(7)-Dehydrogenipinic acid	243.0874 (3)<br>225.0770 (25)	$[M-H]^-$<br>$[M - H_2O]^-$	4.05	$C_{11}H_{16}O_6$	-0.2	225.0767<br>149.0606<br>137.0607	Putatively characterized compound classes (III)
3(4)-Dehydrogenipic acid	185.0819 (4)	$[M-H]^-$	3.89	$C_9H_{14}O_4$	0.3	167.0712<br>155.0713<br>141.0920<br>123.0815<br>93.0708	Putatively characterized compound classes (III)
3(7)-Dehydrogenipinic acid isomer	243.0874 (5)	$[M-H]^-$	4.67	$C_{11}H_{16}O_6$	0.1	225.0767<br>213.0767<br>169.0869<br>149.0606<br>137.0607	Putatively characterized compound classes (III)
8-Hydroxygenipic acid	199.0614 (6)	$[M-H]^-$	4.05	$C_9H_{12}O_5$	1.4	181.0505<br>155.0712<br>153.0553<br>137.0606<br>125.0607<br>111.0815<br>93.0709	Putatively characterized compound classes (III)
Unknown	213.0770 (8)		3.91			181.0506<br>137.0607<br>119.0500<br>95.0502<br>73.0294	(IV)

Table S3. continued

Annotated metabolite	MS Main ions *m/z* (*p*-value classification)	Ion	RT (min)	Proposed precursor ion formula	Mass precision (ppm)	MS/MS (*m/z*) main fragments (30-40 v)	Reference/ identification level
Unknown	185.0456 (10)		3.50			167.0348<br>141.0557<br>125.0969<br>123.0815<br>97.06583	(IV)
Unknown	186.0853 (11)		3.88			168.0747<br>143.0713<br>124.0848<br>94.0742	(IV)
Unknown	227.0563 (12)		2.60			210.0881<br>183.0661<br>165.0555<br>121.0657	(IV)
Unknown	213.0771 (14)		4.34			183.0662<br>137.0607<br>73.0294	(IV)
Genipic acid	183.0664 (15)	$[M-H]^-$	5.39	$C_9H_{12}O_4$	0.9	168.0060<br>155.0348<br>139.0763	HMDB ID: HMDB0036072 (III)
Hydroxymethyl-cyclopenta[c]furan-1,3diol	171.0664 (16)	$[M-H]^-$	2.59	$C_8H_{12}O_4$	0.8	153.0555<br>142.9464<br>109.0657<br>79.0552	Putatively characterized compound classes (III)
Unknown	345.1191 (17)		5.08			327.1081<br>285.0978<br>113.0243<br>75.0086	(IV)
Unknown	197.0820 (18)		6.61			179.0713<br>153.092	(IV)
Unknown	211.0614 (19)		4.23			193.0496<br>181.0507<br>167.0713<br>151.0399	(IV)

Table S3. continued

Annotated metabolite	MS Main ions *m/z* (*p*-value classification)	Ion	RT (min)	Proposed precursor ion formula	Mass precision (ppm)	MS/MS (*m/z*) main fragments (30-40 v)	Reference/ identification level
Unknown	359.0986 (20)		4.79			183.0662<br>175.0248<br>155.0350<br>139.0764<br>113.0244	(IV)
Unknown	213.0772 (21)		4.94			133.0489<br>79.9573	(IV)
Unknown	213.0770 (22)		5.31			195.0665<br>181.0505<br>169.0869<br>151.0764<br>137.0607<br>125.0971<br>119.0502<br>95.0502<br>73.0294	(IV)
Nonate	187.0977 (23)	$[M-H]^-$	4.31	$C_9H_{16}O_4$	1.1	169.0869<br>141.0920<br>125.0972	METLIN ID: 44183 (II)
Unknown	201.0771 (24)		4.42		1.4	183.0663<br>157.0869<br>139.0763	(IV)
Unknown	257.1033 (26)		5.51			221.8424<br>213.1132<br>195.1024	(IV)
Hydroxymandelic acid	167.0351 (27)	$[M-H]^-$	4.81	$C_8H_8O_4$	0.7	123.0450<br>121.0293	HMDB ID: HMDB0000822 (III)
Unknown	227.0928 (28)		5.56			209.1151<br>183.1026	(IV)
Unknown	213.0405 (29)		4.21			137.0608<br>125.0607<br>73.0294	(IV)
Genipic acid isomer	183.0663 (30)	$[M-H]^-$	6.39	$C_9H_{12}O_4$	0.5	169.0010<br>139.0761<br>112.9855	HMDB ID: HMDB0036072 (III)

Table S3. continued

Annotated metabolite	MS Main ions *m/z* (*p*-value classification)	Ion	RT (min)	Proposed precursor ion formula	Mass precision (ppm)	MS/MS (*m/z*) main fragments (30-40 v)	Reference/ identification level
Unknown	217.1083 (31)		4.74			199.0977<br>137.0243<br>93.0345<br>79.9572	(IV)
3-Hydroxy-4-methoxymandelate	197.0456 (32)	$[M-H]^-$	1.70	$C_9H_{10}O_5$	0.4	179.0350<br>169.0506<br>125.0242	HMDB ID: HMDB0029170 (III)
Unknown	123.0451 (33)		4.83			108.0215<br>95.0138<br>79.0552	(IV)
2-isopropylmalic acid	175.0613 (34)	$[M-H]^-$	4.87	$C_7H_{12}O_5$	0.9	157.0505<br>115.0399<br>85.0658	Identified compounds (I)
Unknown	256.9763 (35)		4.86			177.0192<br>137.0606<br>73.0295	(IV)
Unknown	457.1027 (36)		6.31			175.0248<br>145.0618<br>113.0244<br>96.9600	(IV)
Unknown	124.0074 (37)		0.55			106.9807<br>94.9807<br>79.9573	(IV)
Unknown	217.1083 (38)		4.31			199.0974<br>137.0970<br>97.0657	(IV)
Unknown	401.1821 (39)		5.72			383.1702<br>341.1605<br>207.1390	(IV)
2,3-dihydroxybenzoate	153.0195 (40)	$[M-H]^-$	4.59	$C_7H_6O_4$	1.0	137.0352<br>123.0090<br>109.0293	Identified compound (I)

Table S3. continued

Annotated metabolite	MS Main ions *m/z* (*p*-value classification)	Ion	RT (min)	Proposed precursor ion formula	Mass precision (ppm)	MS/MS (*m/z*) main fragments (30-40 v)	Reference/ identification level
Unknown	371.1346 (41)		5.72			353.1236<br>311.1132<br>177.0920	(IV)
3,4-Methylene suberic acid	197.0820 (42)	$[M-H]^-$	6.76	$C_{10}H_{14}O_4$	0.8	179.0721<br>153.0920<br>135.0815<br>59.0137	HMDB ID: HMDB59768 (III)
Unknown	215.0021 (43)		5.04			178.0510<br>153.0920<br>135.0450	(IV)
cis-4-Decenedioic acid	199.0977 (44)	$[M-H]^-$	6.95	$C_{10}H_{16}O_4$	1.0	197.0974<br>181.0869<br>155.1077<br>137.0971	HMDB ID: HMDB0000603 (III)
Unknown	211.0614 (45)		4.75			146.0250<br>132.0453<br>80.9650	(IV)
Unknown	153.0921 (46)		6.74			135.0815<br>124.0164<br>109.0294<br>59.0138	(IV)
Unknown	133.0507 (47)		1.72			115.0397<br>87.0086<br>71.0137	(IV)
Unknown	249.0076 (48)		3.52			169.0141<br>121.0329	(IV)
3,4-Dihydroxyphenylglycol O-sulfate	249.0076 (49)	$[M-H]^-$	4.01	$C_8H_{10}O_7S$	0.6	169.0506<br>151.0395<br>139.0035	HMDB ID: HMDB0001474 (III)

Table S3. continued

Annotated metabolite	MS Main ions *m/z* (*p*-value classification)	Ion	RT (min)	Proposed precursor ion formula	Mass precision (ppm)	MS/MS (*m/z*) main fragments (30-40 v)	Reference/ identification level
Unknowm	377.1454 (50)		6.58			377.1456<br>183.1026<br>201.1132<br>113.0244	(IV)

Levels of metabolite identifications: I, Identified compounds; II, Putatively annotated compounds; III, Putatively characterized compound classes; IV, Unknown compounds.

Figure S1: Common and more specific biomarker of genipap consumption at each time-range. The intersection indicates the steadiest biomarkers across each time-range. Multiplex Pred indicates the predictive multiplex biomarker.

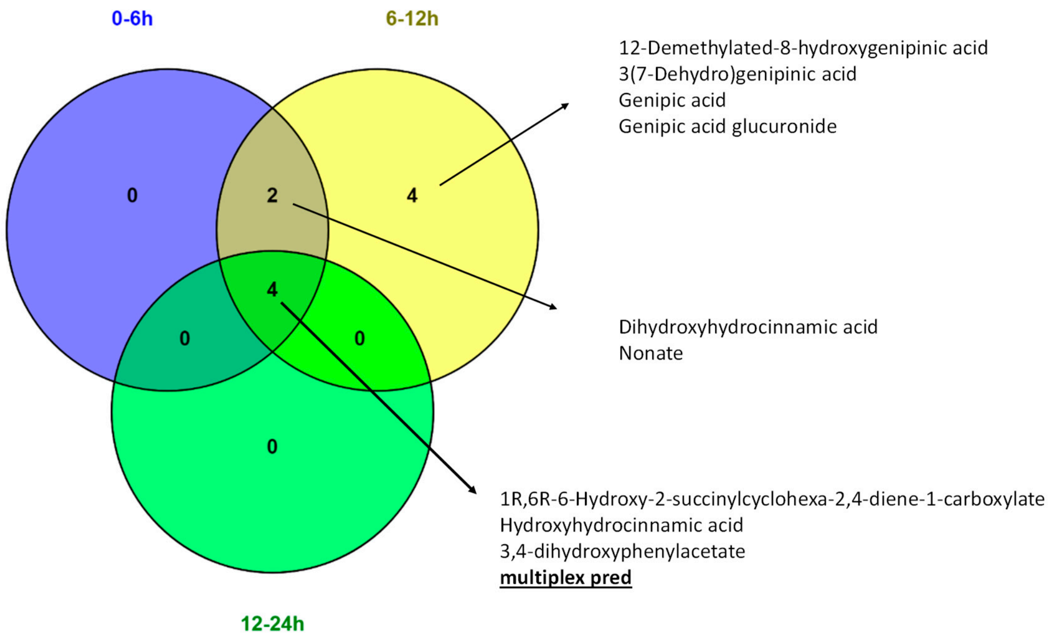

Figure S2: Genipap consumers' vs non-consumers' prediction using the urine multiplex biomarkers measured at 0-6h, 6-12h and 12-24h. The cut off values indicating genipap consumption status were permuted to assess the impact of the sampling time on the prediction over 24h. The actual cut off values of each time-range are indicated in red. Misclassified samples are indicated with an arrow.

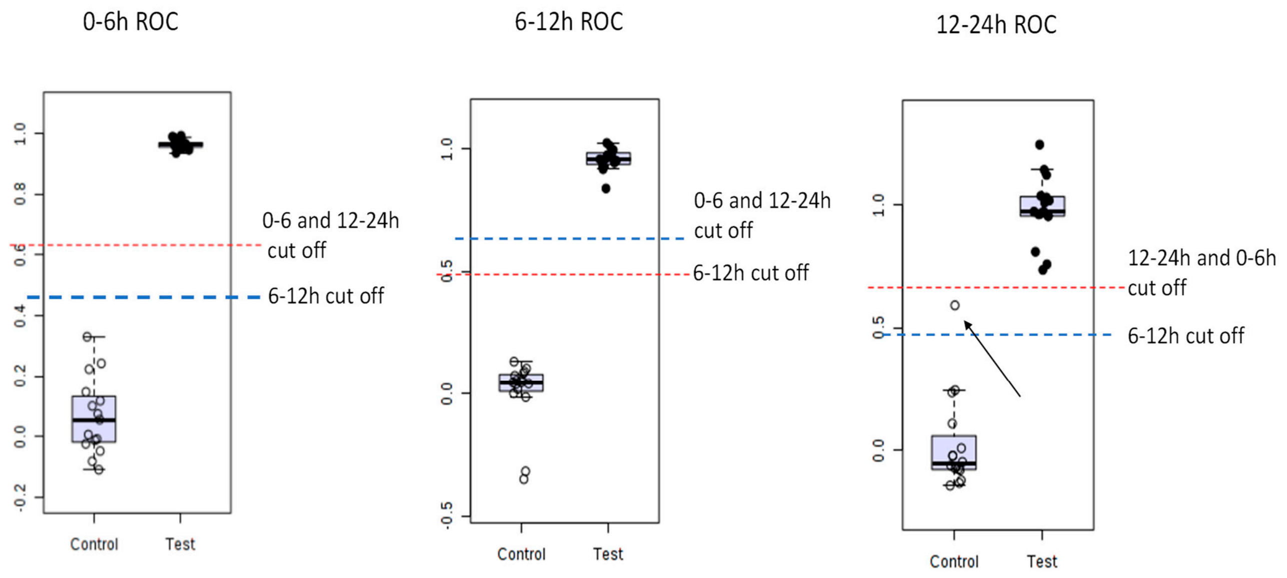

Figure S3: Interindividual variation of best predicting metabolites (ROC AUC of 1, Table 2) at 0-6h (A), 6-12h (B), and 12-24h (C) urine compared to the multiplex biomarker scores (yellow line) calculated over the same time ranges. All values are scaled to 1 to ease comparisons.

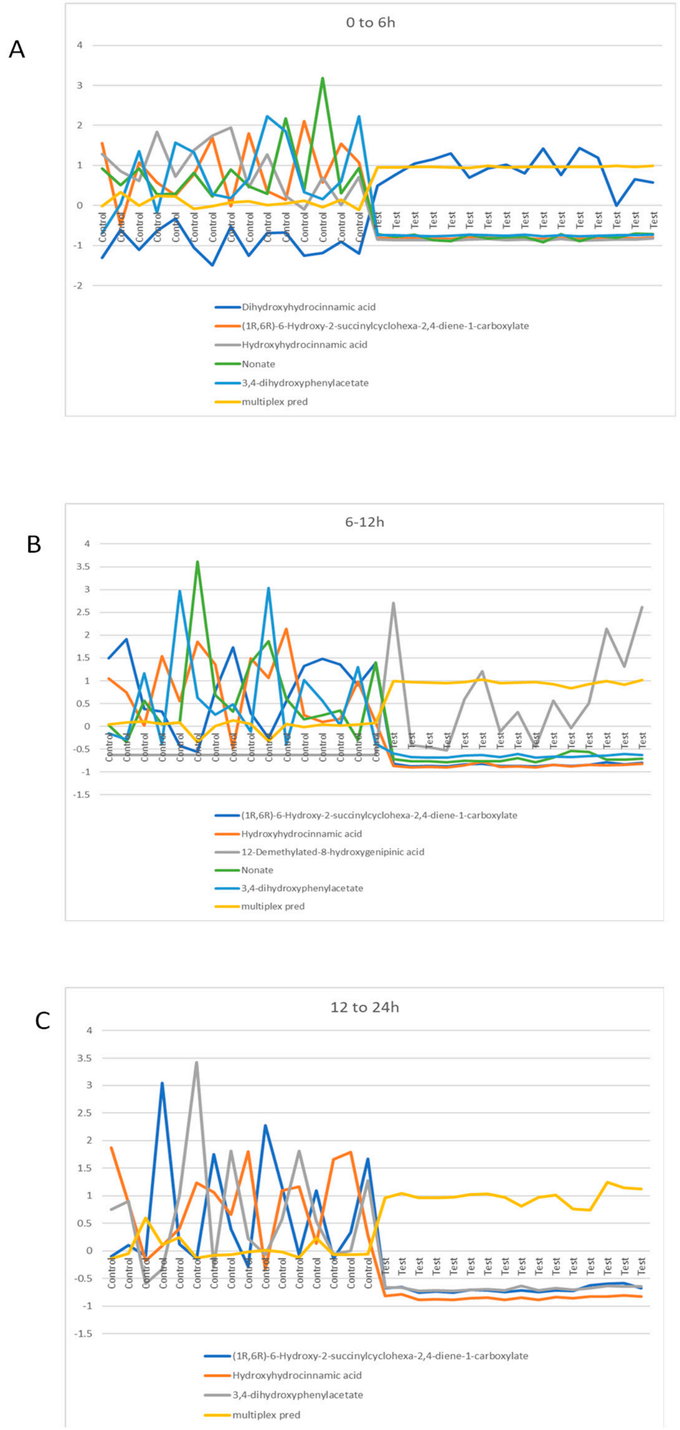

Figure S4: MS/MS spectra fragmentation. (A) spectrum of commercial standard of genipin, (B) spectra of a metabolite obtained from human urine sample after drink genipap juice ([M + H]+, *m/z* 227.0911, retention time: 4.04).

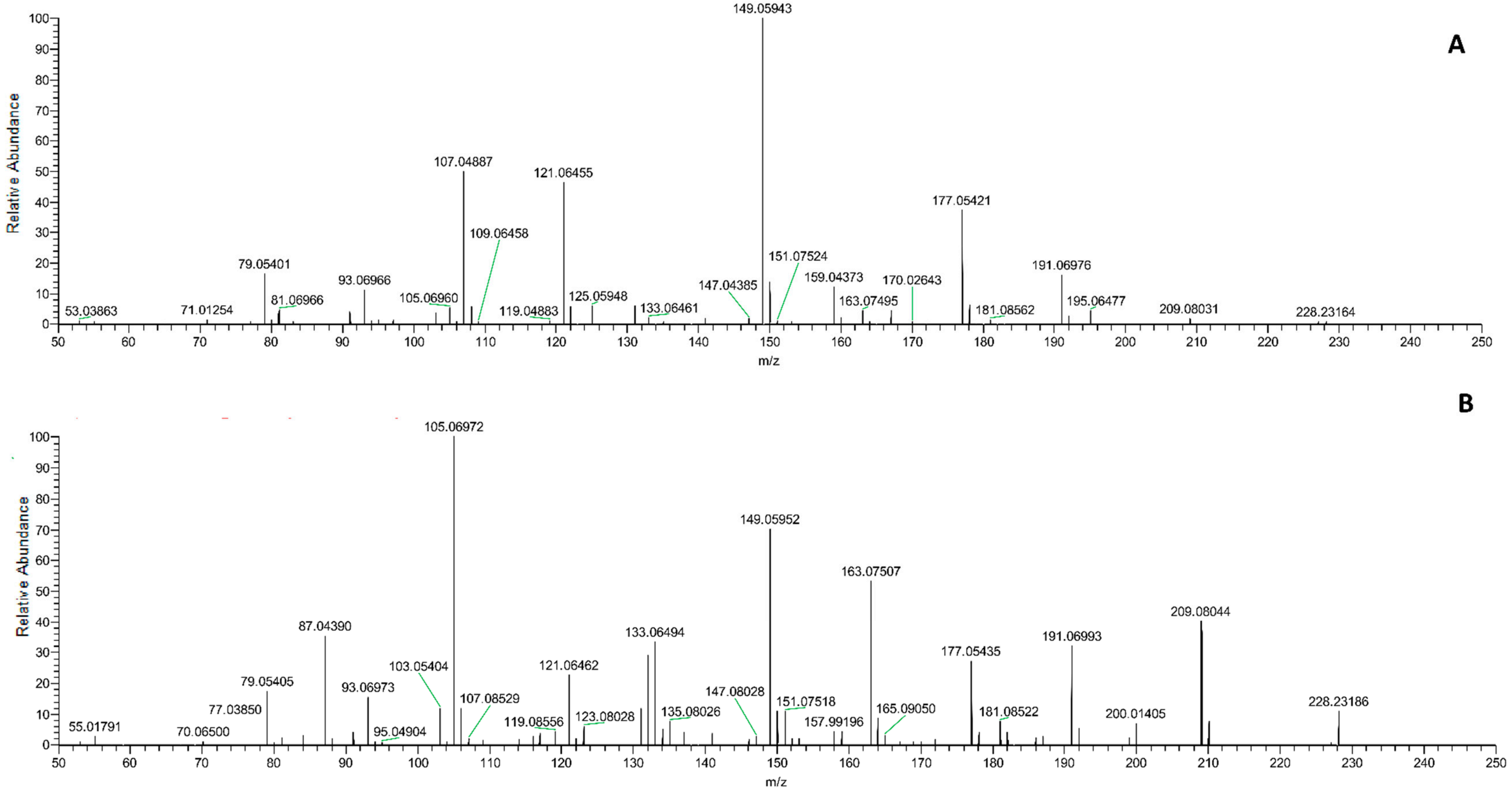

Supplement: Supplementary file 1 [file nutrients-10-01155-s001.pdf]
